# A System of Dental Surgery

**Published:** 1898-07

**Authors:** 


### Bibliography.

A System of Dental Surgery. By the late Sir John Tomes, F.R.S. Fourth Edition, revised and enlarged by Charles S. Tomes, M.A., F.R.S. With two hundred and eighty-nine illustrations. Philadelphia: P. Blakiston, Son & Co.

Tomes's "Dental Surgery," long recognized as a standard work, comes to us much improved by revision and intelligent editing. Considerable obsolete matter has been stricken out, its place being well filled with principles and methods of more recent origin. It is one of the works that should be found in the library of every dentist or physician who attempts to keep abreast with the advance in dental surgery.

Several of the chapters, however, could yet be reduced to advantage, and the work would be much improved were it well illustrated. The editor, too, does not always make his subject sufficiently clear for students' use. For instance, under erosion of the teeth he cites several cases, showing that dental erosion is a fact, but does not offer a satisfactory hypothesis as to the cause of this lesion. Later investigations, however, indicate that etiology of dental erosion is the result of the action of acid secretions from the labial glands, caused, probably, by the patient being of the uric acid diathesis. In some cases, where these glands become diseased or enlarged, Professor Brubaker is on record as recommending their destruction by the electric needle. But none of this is referred to by Mr. Tomes. Under methods of filling carious teeth, retaining points or pits are recommended for anchorage instead of the groove or undercut.

These imperfections, we trust, may be rectified in future editions; but, all in all, the work is a distinct fulfilment of the author and editor's useful aims.

